# Ancient Components and Recent Expansion in the Eurasian Heartland: Insights into the Revised Phylogeny of Y-Chromosomes from Central Asia

**DOI:** 10.3390/genes13101776

**Published:** 2022-10-01

**Authors:** Maxat Zhabagin, Lan-Hai Wei, Zhaxylyk Sabitov, Peng-Cheng Ma, Jin Sun, Zhanargul Dyussenova, Elena Balanovska, Hui Li, Yerlan Ramankulov

**Affiliations:** 1 National Center for Biotechnology, Nur-Sultan 010000, Kazakhstan; 2 School of Sciences and Humanities, Nazarbayev University, Nur-Sultan 010000, Kazakhstan; 3 Social and Human Evolution Laboratory, School of Ethnology and Anthropology, Inner Mongolia Normal University, Hohhot 010022, China; 4 B&R International Joint Laboratory for Eurasian Anthropology, School of Life Science, Fudan University, Shanghai 200438, China; 5 L. N. Gumilyov Eurasian National University, Nur-Sultan 010000, Kazakhstan; 6 Research Institute for Jochi Ulus Studies, Nur-Sultan 010000, Kazakhstan; 7 School of Life Sciences, Jilin University, Changchun 130012, China; 8 Xingyi Normal University for Nationalities, Xingyi 562400, China; 9 Research Centre for Medical Genetics, 115552 Moscow, Russia; 10 MOE Key Laboratory of Contemporary Anthropology, School of Life Sciences, Fudan University, Shanghai 200438, China

**Keywords:** Central Asia, Y-chromosome, paternal lineage, admixture

## Abstract

In the past two decades, studies of Y chromosomal single nucleotide polymorphisms (Y-SNPs) and short tandem repeats (Y-STRs) have shed light on the demographic history of Central Asia, the heartland of Eurasia. However, complex patterns of migration and admixture have complicated population genetic studies in Central Asia. Here, we sequenced and analyzed the Y-chromosomes of 187 male individuals from Kazakh, Kyrgyz, Uzbek, Karakalpak, Hazara, Karluk, Tajik, Uyghur, Dungan, and Turkmen populations. High diversity and admixture from peripheral areas of Eurasia were observed among the paternal gene pool of these populations. This general pattern can be largely attributed to the activities of ancient people in four periods, including the Neolithic farmers, Indo-Europeans, Turks, and Mongols. Most importantly, we detected the consistent expansion of many minor lineages over the past thousand years, which may correspond directly to the formation of modern populations in these regions. The newly discovered sub-lineages and variants provide a basis for further studies of the contributions of minor lineages to the formation of modern populations in Central Asia.

## 1. Introduction

The demographic history of Central Asia, the Eurasian heartland, has long fascinated linguists, archaeologists, and geneticists. The genomes of ancient human individuals from Ust’Ishim, Mal’ta, and Yana sites in northern Eurasia suggest that Central Asia is a route for the dispersal of *Homo sapiens sapiens* from the Middle East to northern Eurasia [1,2,3]. The Last Glacial Maximum (LGM) substantially reshaped the distribution of ancient human populations in Central Asia and adjacent regions [4,5,6,7,8]. Several studies of ancient DNA have focused on the genetic structure of ancient populations before and after the rise of Neolithic farmers and Indo-European populations in Central Asia [9,10,11,12,13,14,15,16]. The ancient Botai people [9] may be genetically related to populations in Ancient Northern Eurasia, as revealed by studies of a 24,000-year-old Mal’ta boy and related ancient remains [2]. Neolithic farmers diffused in all directions from the Middle East and established the initial Neolithic archaeological cultures in the Indus Valley and the southern part of Central Asia [11]. About 5000 years ago, Indo-European-speaking populations arose in the western part of the Eurasian steppe. The eastward diffusion of Indo-European-like populations led to the emergence of the Chalcolithic Age and Bronze Age across Central Asia and the eastern Eurasian steppe [9,10,11,12,13,14]. It is generally accepted that the Andronovo culture complex was created by the ancestors of Indo-Iranian-speaking populations [17]. The later rise of Scythians triggered large-scale migration and admixture across the Eurasian steppe, including Central Asia [12,15].

The trend towards the diffusion of human populations from west to east shifted about 1200 years ago. The Karasuk culture arose in South Siberia and expanded across a vast geographical area, giving rise to a series of successors [18]. In a later historical period, Yuezhi, Usuns, and Ephthalite people migrated from east to west and established a series of polities in Central Asia [19]. Since the beginning of the Christian era, the ancient Xiongnu/Huns moved westward on a large scale and significantly changed the ethnic pattern in Central Asia and eastern Europe [20]. Later, ancient Turkic and Mongolic tribes spread westward and scattered throughout Central Asia, the Middle East, and Eastern Europe [21]. The descendants of the above-mentioned ancient people mixed and formed the modern populations in Central Asia over a long historical period [22,23,24].

Previous studies of modern samples have shed light on the origin and genetic structure of populations from Central Asia [25]. Ancient DNA analyses of remains from various historical periods have revealed the genetic structure of ancient people and their contribution to the gene pool of modern populations as well as patterns of admixture [9,10,11,12,13,14]. In general, paternal, maternal, and autosomal gene pools of most populations from this region are characterized by high diversity and admixture with migration from the Middle East, South Asia, East Asia, Northern Asia, and Europe.

Previous research has also focused on the formation of modern populations in the recent millennium [23,24]. A strong tribe structure has been detected among populations from this region [26]. The tribal tradition leads to great differences in dominant paternal lineage between tribes [27,28,29,30,31,32,33,34,35,36]. By contrast, within a tribe, paternal Y-chromosomal short tandem repeat (Y-STR) diversity is generally low [34]. Although gene flow is common, the genetic relationships among populations are still closely related to the language category. For example, the Tajik people are genetically related to other Indo-European-speaking populations [28,30,37], while many genetic components from Siberia have been detected in the Kyrgyz people [38]. In addition, Y-chromosome sequences of specific tribes or family clans have been evaluated to explore the formation of related populations [27,32,33]. Nevertheless, the demographic histories of populations from Central Asia are complex, and more work is needed for a clearer understanding.

In this study, we analyzed 187 Y-chromosome sequences of populations from Central Asia. Our first objective was to reveal general patterns in the paternal gene pool based on full-length sequences. Our second objective was to identify paternal lineages that are nearly unique to a specific population, and to analyze the demographic history. Specifically, we evaluated the different distinct periods of genetic exchange and divergence among ancient populations in Central Asia.

## 2. Materials and Methods

**Figure 1:**
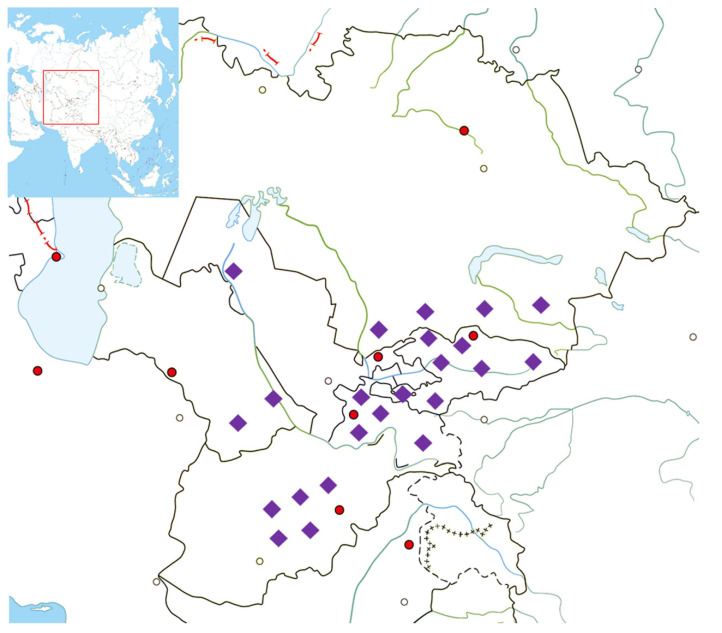
Approximate sampling locations. Detailed information is provided in Table S1. Purple rhombi indicate the sampling locations. Red and transparent circles indicate the capitals and major cities of countries, respectively.

Saliva samples were collected from unrelated healthy males from populations in Central Asia. All participants provided signed consent prior to participating in the study. The present study complies with the ethical principles of the 2013 Helsinki Declaration of the World Medical Association. The Local Ethical Commission of at the National Center for Biotechnology and Nazarbayev University Institutional Research Ethics Committee approved the study. DNA specimens extracted from 187 samples were sent for next-generation sequencing using the Illumina HiSeq2000 platform (San Diego, CA, USA). The target depth was 10× and data were sufficient for analyses. The sample set included 20 Kyrgyz, 20 Uzbeks, 17 Karakalpaks, 20 Hazara, 9 Karluks, 20 Tajiks, 19 Uyghur, 20 Dungan, 20 Turkmen, and 22 Kazakhs from Kazakhstan. Figure 1 shows the places of origin of individuals’ paternal ancestors, which are the historical homelands of their grandfathers and fathers in Afghanistan, Uzbekistan, Kyrgyzstan, Turkmenistan and Tajikistan. Detailed sample information can be found in Supplementary Table S1.

Data analysis: Read mapping and SNP calling from next-generation sequencing data were conducted using standard procedures (BWA and SAMtools) and the human reference genome sequence hg38 [39,40]. Bayesian evolutionary analyses were conducted using BEAST (v.2.4.3) [41]. To calculate divergence times in the phylogenetic tree, a point mutation rate of 0.74 × 10^−9^ per site per year was applied [42], which was very similar to the rate of 0.78 × 10^−9^ per site per year reported in analyses of the ancient genome of the 45,000-year-old Ust’Ishim male [1]. We refer to the protocol used in our previous publications for data processing and Bayesian evolutionary analyses [7,43,44]. The details of data analysis can be found in the Supplementary Text.

## 3. Results

**Figure 2:**
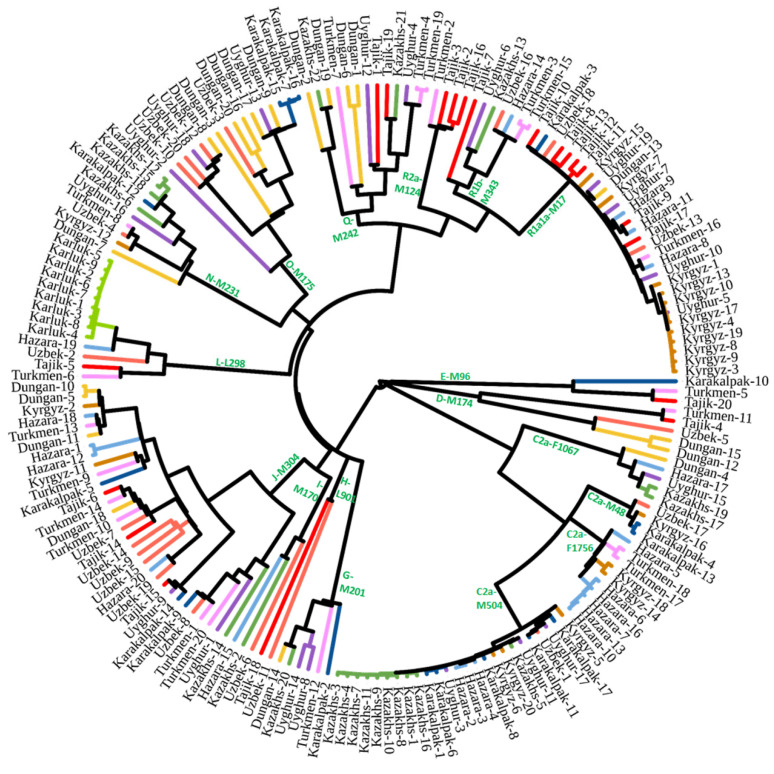
Revised phylogenetic tree based on Y-chromosomes of populations in Central Asia.

We used Y-chromosome sequences of 187 males to construct a revised phylogenetic tree (Figure 2; Table S1). The detailed tree can be found in Figure S1. The frequencies of Y-chromosome haplogroups in the studied populations are shown in Table 1. Haplogroup C2a1a1b1-F1756 was the predominant paternal lineage of the ancient Dong-Hu and Xian-Bei tribes [45,46,47]. The appearance of this lineage among Hazara, Kyrgyz, and Turkmen indicated that these populations originated in Eastern Eurasia. Haplogroup C2a1a2a-M48-F6379 had a high frequency in the Junior Juz of Kazakh and Oirat people, which are western Mongolic-speaking populations [32]. The appearance of this lineage among the Karakalpak suggested that this population is closely related to Kazakhs and ancient Mongols. It is thought that the expansion in Central Eurasia of C2a1a3-M504 is directly related to the activity of ancient Mongol tribes in the past millennium [26,48,49]. The high frequency of this lineage in the Hazara, Karakalpak, Kazakh, Kyrgyz, and Uyghur populations in this study is consistent with the proposed origin of these populations and previous research [24,48]. The downstream lineages of N-M231 among Kazakhs (Tables S1 and S2) are related to ancient Turkic tribes. D-M174 and O-M175 detected in populations from Central Asia resulted from admixture with populations from East Asia over the past 2000 years.

Consistent with previous research, as shown in Table 1, haplogroups E-M96, G-M201, H-L901, I-M170, and L-M20 were widely distributed with low frequencies in populations from Central Asia. These lineages represent admixture from South Asia, the Middle East, and Europe. Nevertheless, we found a special lineage, L-M20, in the Karluks. Nine samples of this lineage from the Karluks were sequenced. We found a unique sub-branch, L1a2a-M2398-Y236528, in these samples; this lineage separated from the lineages found in India about 1500 years ago (Tables S1 and S2, Figure S1).

**Figure 3:**
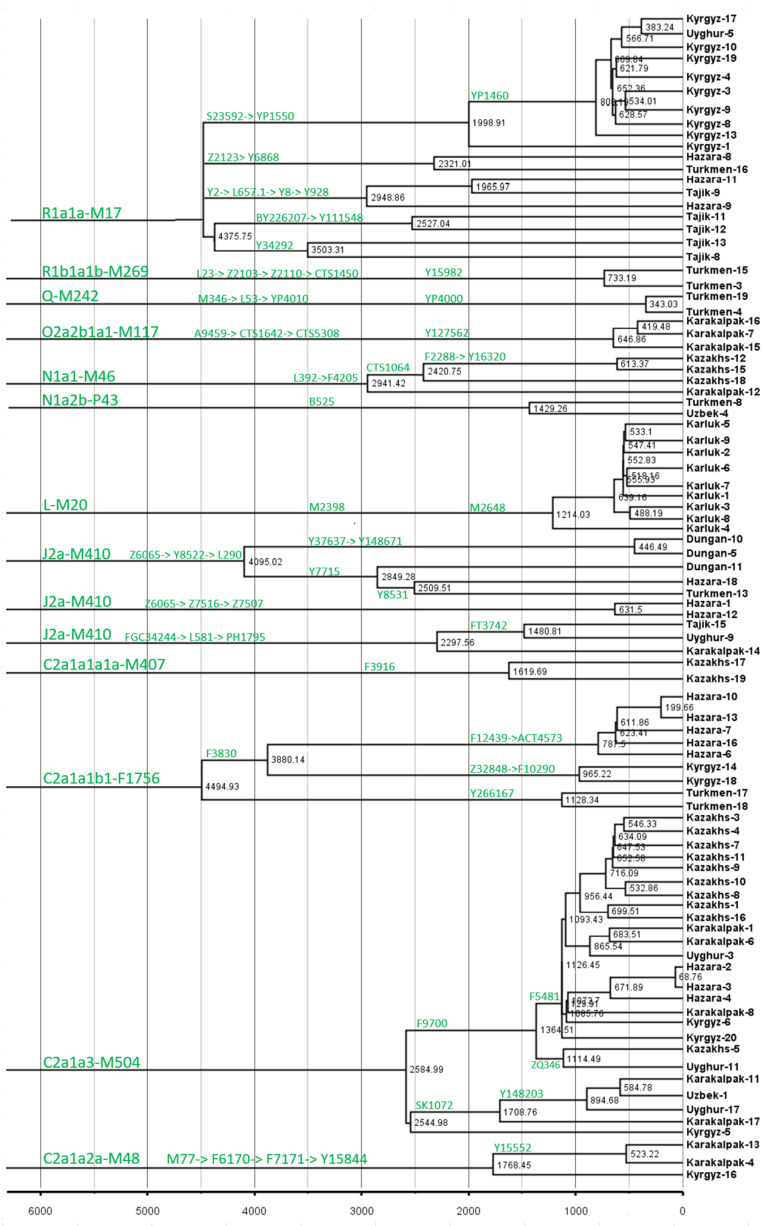
Schematic tree of unique minor lineages in populations from Central Asia.

Previous studies have demonstrated that South Siberia is likely the center of diffusion of haplogroup Q-M242 beginning 30,000 thousand years ago [6,50,51]. Furthermore, many minor sub-lineages of Q-M242 have been detected in populations from Inner Eurasia, including Mongolic- and Turkic-speaking populations [6,23,50,51]. We detected diverse sub-lineages of Q-M242 (Table S1). As revealed by previous studies, R1a1a-M17 and R1b-M343 are two major paternal lineages of Indo-European-speaking populations [52,53]. Most modern populations in Central Asia still show a high frequency of R1a1a-M17 (Table 1). The haplogroup R2a-M124 was widely found in South Asia [25,54]. The diffusion of this lineage in Central Asia and the Mongolian Plateau has been unclear. The revised high-resolution phylogenetic tree with age estimation provides a detailed overview of paternal gene flow in Central Asia populations (Table 1, Figure 2, Figure 3 and Figure S1). The high diversity of macro-haplogroup lineages suggested that there was intense admixture from different peripheral areas of the Eurasian continent, like C/D/N/O from Eastern Eurasia, E/J from the Middle East, H/L from South Asia, I/R1 from Western Eurasia, and Q from South Siberia.

The most important finding of this project is the identification of unique minor lineages that experienced a recent expansion in Central Asia (Figure 3 and Figure S1, Table S2). We compared the results of these study with data from other sources, like ancient DNA [11,12,13,14,15,16] (Table S3) and a publicly available phylogenetic tree of global humans from https://www.yfull.com/tree (accessed on 1 September 2022) and https://www.23mofang.com/ancestry/ytree/root (accessed on 1 September 2022). We identified the closest branch to these minor lineages with a recent separation time among populations from peripheral areas of Eurasia. As shown in Figure 3, we identified tens of minor lineages (also see Figure S1), including 33 minor lineages with ages younger than 2000 years.

## 4. Discussion

### 4.1. Multiple Layers of Deposition of Ancient Components

Central Asia is not a region of origin of agriculture in the Neolithic age or technological systems of the Bronze Age. Therefore, the first wave of population growth caused by these technological revolutions did not occur in Central Asia. Genetic studies generally support the results of studies in other disciplines. Available genetic data indicate that the sources of populations in Central Asia include all peripheral regions of Eurasia, including South Asia, the Middle East, Europe, North Asia, and East Asia. This general scenario is consistent with the geographical location of Central Asia as the heartland of Eurasia.

We also observed multiple layers of deposition of genetic components of ancient populations. The Neolithic evolution in the Middle East led to the global expansion of haplogroup J-M172 and related haplogroups [55]. Ancient DNA studies suggest that the paternal haplogroup J-M172 from the Middle East led to the appearance of the initial Neolithic settlements in the southern part of Central Asia [11]. In a later historical period, continuous migration from the Middle East also brought more sub-branches of J-M172. Many ancient DNA studies have shown that the expansion of paternal R1a1a-M17 and R1b-M269 is consistent with the spread of Indo-European populations [16,56,57,58], and this may be related to the diverse sub-branches of R1a1a-M17 in Central Asia. In addition, G-M201 is also likely derived from Indo-European populations. Based on previously reported ancient DNA and modern population data, we speculate that some lineages downstream of Q-M242, N1a1-M46, R1a1a-M17, and C2a1b1-F1756 in Central Asia could be attributed to migration by Turkic people. Finally, referring to previous literature, we speculate that the spread of Mongolian populations introduced C2a1a3-M504, C2a1b1-F1756, and sub-branches of C2a1a2a-M48 to Central Asia. During East-to-West migration over thousands of years, many paternal haplogroups in East Asia spread to Central Asia, including several sub-branches of D-M174, C2a-F1096, and O-M175. In short, due to its high mutation rate, the Y chromosome easily forms population-specific sub-branches. Therefore, the main haplogroups differ among populations from different regions in the periphery of Eurasia. Based on an analysis of the complete Y chromosome sequence, we can identify patterns of large-scale population diffusion in different periods from the complex genetic structure of the population across Central Asia.

### 4.2. Recent Expansion and the Formation of Modern Populations

We predicted that the recent consistent expansion of minor lineages contributed directly to the formation of modern populations in Central Asia. The main paternal haplogroups of populations in different regions of Eurasia include C-M217, D-M174, E-M96, G-M201, H-L901, I-M170, I-M304, L-L298, T-M184, N-M231, O-M175, Q-M242, and R-M207. These macro-haplogroups are common in Central Asian populations; however, the frequencies vary substantially. At the sub-branch level, Central Asian populations have specific dominant haplogroups. As shown in Figure 3, there were many ethnicity-specific minor lineages among populations in Central Asia, such as R1a-MM17-Y34292, and R1a-M17-Y111548 of the Tajiks. We also observed many sub-branches that expanded in the last 1000 years, such as C2a1a3-M504-F5481, L-M20-M2648, and R1a1a-M17-YP1460. Many minor lineages of R1a1a-M17 in the Tajik people are related to Indo-European ancestors. Additionally, many minor branches that expanded in the past 1000 years were observed in the Kazakh, Kyrgyz, Qarluk, Karakalpak, and Hazara. In conclusion, we believe that the expansion of these specific lineages is directly related to the formation of modern ethnic groups in Central Asia. The differentiation of different paternal lineages in different periods led to population increases and laid the foundation for modern Central Asian populations.

In summary, we constructed a high-resolution phylogeny of paternal lineages for populations from Central Asia. We detected high haplogroup diversity among populations. Admixture from all peripheral areas of the Eurasian continent was observed among the paternal gene pool of these populations. Further phylogeny analyses revealed many minor lineages specific to populations from Central Asia and expansion events in the past 3000 or 1000 years. We proposed that the multiple large-scale demographic events in different historical periods of Central Asia left clear layers of deposition of ancient components in the gene pool of modern populations. More importantly, the expansion of minor lineages observed in this study may correspond to the formation of modern populations in Central Asia in the past thousand years. More work is needed to explore the detailed demographic history in Central Asia.

## Figures and Tables

**Table 1:** Frequencies of Y-chromosome haplogroups in various populations. Gray shading indicates the data that had a frequency higher than 10%.

Haplogroup	Dungan	Hazara	Karakalpak	Karluk	Kazakhs	Kyrgyz	Tajik	Turkmen	Uyghur	Uzbek	Sum
C2a1a1b1-F1756		6 (0.3)				2 (0.1)		2 (0.1)			**10**
C2a1a2a-M48			2 (0.12)			1 (0.05)				1 (0.05)	**4**
C2a1a3-M504		3 (0.15)	5 (0.29)		10 (0.45)	3 (0.15)			3 (0.14)	1 (0.05)	**25**
C2b*-F1067xM407	1 (0.05)	1 (0.05)									**2**
C2b1a1a1a-M407					2 (0.09)				1 (0.05)		**3**
D1a1a2-N1	2 (0.1)									1 (0.05)	**3**
D1a2a-P47							1 (0.05)	1 (0.05)			**2**
E1b1b1-M35.1			1 (0.06)				1 (0.05)	1 (0.05)			**3**
G-M201	1 (0.05)		1 (0.06)		1 (0.45)			1 (0.05)	2 (0.09)		**6**
H-L901							1 (0.05)			1 (0.05)	**2**
I-M170		1 (0.05)			1 (0.45)					1 (0.05)	**3**
J1a-CTS5368			1 (0.06)		1 (0.45)			2 (0.1)	1 (0.05)	1 (0.05)	**6**
J2a-M410	4 (0.2)	4 (0.2)	2 (0.12)			2 (0.1)	3 (0.15)	4 (0.2)	1 (0.05)	5 (0.25)	**25**
L-M20		1 (0.05)		9 (1)			1 (0.05)	1 (0.05)		1 (0.05)	**13**
N-M231	1 (0.05)		1 (0.06)		4 (0.18)	1 (0.05)		1 (0.05)	1 (0.05)	1 (0.05)	**10**
O-M175	6 (0.3)		3 (0.18)						3 (0.14)	4 (0.2)	**16**
Q-M242	4 (0.2)				2 (0.09)		2 (0.1)	3 (0.15)	2 (0.09)		**13**
R1a1a-M17	1 (0.05)	3 (0.15)	1 (0.06)			11 (0.55)	7 (0.35)	1 (0.05)	4 (0.18)	2 (0.1)	**30**
R1b-M343		1 (0.05)			1 (0.45)		1 (0.05)	2 (0.1)	1 (0.05)	1 (0.05)	**7**
R2a-M124							3 (0.15)	1 (0.05)			**4**
**Sum**	**20**	**20**	**17**	**9**	**22**	**20**	**20**	**20**	**19**	**20**	**187**

## Data Availability

Following the regulations of the Ethical Commission of at the National Center for Biotechnology, the data that support the findings of this study are available on request from the corresponding author. A detailed list of variants is provided in Supplementary Table S2; this list is sufficient for the replication of genetic analyses in this study.

## Supplementary Materials

The following supporting information can be downloaded at: https://www.mdpi.com/article/10.3390/genes13101776/s1. Table S1: Detailed information on the studied samples. Table S2: List of variants of Y-chromosome sequences used in the study. Figure S1: Detailed phylogenetic tree of the studied samples. Table S3: Y-chromosomal information of ancient individuals from Central Asia and adjacent regions. Supplementary Text: Details of data analysis. The references [59,60,61,62,63,64] are cited in the Supplementary Materials.
