# Supplementary material for: Ancient Components and Recent Expansion in the Eurasian Heartland: Insights into the Revised Phylogeny of Y-Chromosomes from Central Asia
Source: Genes (Basel). 2022 Oct 1;13(10):1776. doi: 10.3390/genes13101776 (PMC9601478; doi:10.3390/genes13101776)
Supplement: Supplementary file 1 [file genes-13-01776-s001.zip › genes-1859371-supplementary.pdf]

Supplementary Text

Details of data analysis 1
Generating data of Y-chromosome sequences 1
SNP calling 1
Determine the variant list 2
Bayesian evolutionary analyses 3

Details of data analysis

Generating data of Y-chromosome sequences

Read mapping and SNP calling from next-generation sequencing data were conducted using standard procedures (BWA and SAMtools) and the human reference genome sequence, hg38 [40, 59]. Firstly, the raw data (*id.fq* file)were mapped to reference genome sequence to generate *id.sam* and *id.bam* file with index. Then the Y-chromosome sequence were extracted from the whole genomes and generate the *id.y.bam* file. Such files can be used to combine with data from other resources.

SNP calling

Starting from *.fq* file or *.bam* file, the standard steps for SNP calling can be done with the following commands:

bwa mem reference.fa ID.fq > ID.sam

```
samtools view -bt reference.fa.fai ID.sam > ID.bam
samtools sort ID.bam ID.sort
samtools rmdup ID.sort.bam ID.sort.dedup.bam
samtools index ID.sort.dedup.bam
samtools view -bh ID.sort.dedup.bam Y > ID.Y.bam
samtools index ID.Y.bam
samtools mpileup -guSDf reference.fa ID.Y.bam | bcftools view -cvNg - > ID.Y.vcf
```

Then combine the *.vcf* files of all studied samples into one single .vcf file. This file can be used for the following analysis steps.

Determine the variant list

To obtain a confident Y-SNP dataset for reconstruction of phylogenetic tree and age estimation, we applied a series of strict filters on the original variants file (.vcf file), including: 1, restriction to variants that are single nucleotide polymorphisms (Y-SNP); 2, removal of sites with <80% call rate on all sequences; 3, removal of sites with >5% heterozygous calls on all samples; 4, base coverage ≥3, base quality >20, and distance between SNPs >10 bp; 5, removal of recurrent or triadic variants. The operation above can be done by Excel software or on data processing package of R software.

Since the Non-recombining portion of the Y-chromosome pass down from a father to his son strictly, the SNP on studied region of Y-chromosome follow the strict rule of upper stream and downstream. This means that if a sample share a unique SNP with another sample and form a downstream sub-branches, it is confident that this sample

will contain other mutations that are determined to be upper stream one toward the root of the whole tree. In such case, manual back imputation is an option to generate a clear variant list which will benefit the construction of phylogenetic tree. However, since a haplogroup usually determined by a series of equivalent SNPs, missing of one or two SNPs will not affect the determining of downstream location of a sample on the final phylogenetic tree. Therefore, the back imputation operation is not definitely needed. The BEAST software can handle most of the case and generate a correct tree.

The results of SNP calling and re-arrangement of SNP were showed in Table S2.

We follow the haplpgroup name of human Y-chromosome phylogenetic tree on www.isogg.org. The regulations proposed by the YCC were followed to revise the phylogenetic tree with respect to new splitting branches and new variants in the non-recombining region of the Y chromosome [60] and these regulations are also applied on www.isogg.org.

Bayesian evolutionary analyses

Bayesian evolutionary analyses were conducted using BEAST software (v.2.4.3) [41]. A *.fasta* file generated from variant list can be used as input file for BEAST software. We selected a Bayesian skyline coalescent tree prior and a strict clock. We also applied bModelTest package which allows the BEAST program to infer the most optimized substitution model for input sequences [61]. To calculate splitting times for the phylogenetic tree, we applied a point mutation rate of 0.74×10^{-9} per site per year, which was inferred from the ~12,000-year-old Anzick-1 male infant genome [62].

This rate is quiet close to the value estimated by the ancient genomes of ~45,000-year-old Ust'-Ishim from western Siberia [1]. A generation time of 30 years was used. Both runs were performed with 10 million iterations and sampling every 1 000 steps. Results were visualized using Tracer v.1.6 [63] and FigTree v.1.4.2 [64], with a burn-in of 30% and an effective sample size > 200. The Bayesian evolutionary analyses provided a phylogenetic tree and age of splitting point of the whole tree. These data were used to generate Figure 2, Figure 3, and Figure S1.
